# Supplementary material for: Hopfions emerge in ferroelectrics
Source: Nat Commun. 2020 May 15;11:2433. doi: 10.1038/s41467-020-16258-w (PMC7229001; doi:10.1038/s41467-020-16258-w)
Supplement: Supplementary file 1 — Supplementary Information [file 41467_2020_16258_MOESM1_ESM.pdf]

SUPPLEMENTARY INFORMATION
for:
Hopfions emerge in ferroelectrics

I. Luk'yanchuk,[1] Yu. Tikhonov,[1,2] A. Razumnaya,[2] and V. M. Vinokur[3]

[1]*University of Picardie, Laboratory of Condensed Matter Physics, Amiens, 80039, France.*
[2]*Faculty of Physics, Southern Federal University, 5 Zorge str., 344090 Rostov-on-Don, Russia.*
[3]*Materials Science Division, Argonne National Laboratory, 9700 S. Cass Avenue, Argonne, Illinois 60637, USA.*

SUPPLEMENTARY FIGURES

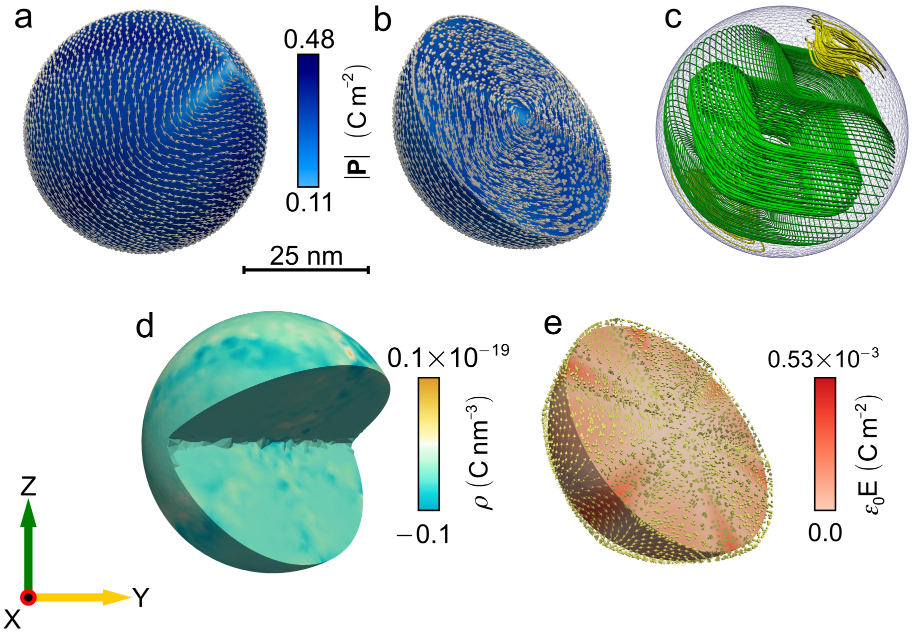

Supplementary Figure 1: The details of the Hopfion formation in the R = 25 nm PZT particle. a, The distribution of the polarization vector, **P**(**r**), over the surface of the nanoparticle. The colour legend shows the amplitude of the polarization. The visible configuration resembles the configuration of the vortex having 3-fold symmetry and with the axis oriented along the [111] crystallographic direction. **b,** The distribution of the polarization vector at the equatorial cut of the nanoparticle. The color legend is the same as in the panel **a**. A cursory look at the texture could have given an impression that this vortex have the structure of the six flux-closure domains, however a careful inspection of the polarization lines reveals the Hopfion structure shown in the next panel. **c,** The Hopfion shown in green lines occupies the most of the interior of the particle. In addition, two polarization vortices, shown in yellow, form in the near vicinity of the poles. **d,** The spatial distribution of the depolarization charge $\rho = \mathrm{div}\,\mathbf{P}$. **e,** The distribution of the depolarization field induced by the depolarization charge from panel **d**. The colour legend shows the amplitude of the electric field, measured in the units of the polarization, which is vanishingly (over three orders of magnitude) small in comparison to the the typical value of the depolarization field $\varepsilon_0 E_\mathrm{d} \simeq P \simeq 0.5\,\mathrm{C\,m^{-2}}$. Panels **d** and **e** demonstrate that the actual depolarization charge in anisotropic nanoparticles acquires a negligibly small value which does not disturb the topological structure of the formed Hopfion.

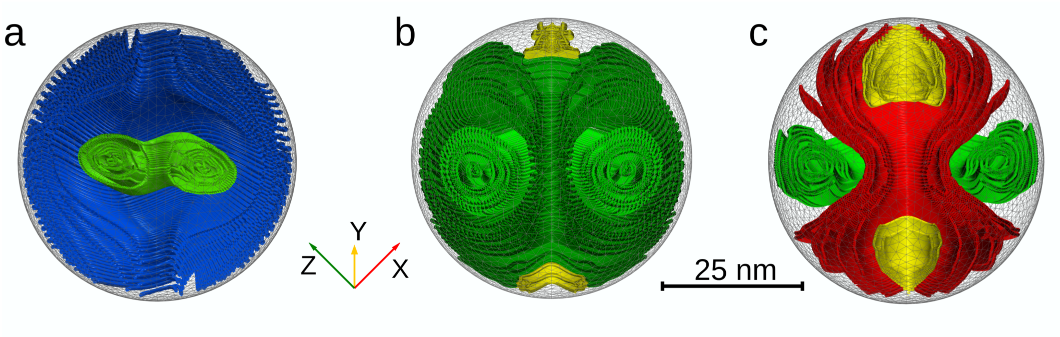

Supplementary Figure 2: Arnold's partitioning of the R = 25 nm PZT particle in different applied fields. The polarization lines are entwined around the nested sets of either cylindrical or toroidal surfaces. The panels show the stages of fibration corresponding to the states (iv), (i) and (v) of the Fig. 3 of the main text. The helices, entwined around the nested cylindrical surfaces with the upward and downward polarization flux are shown in red and blue, respectively; the green colour depicts the localized Hopfion polarization lines, and yellow denotes the closed vortices. **a,** The down-directed field. A cavity contains the toroidal Hopf fibration located at the center of the nanoparticle. The helical states carrying the average polarization flux bypass the Hopfion from the outside. **b,** The zero field. The Hopfion fills up the entire interior of the particle, whereas two closed vortices are located at the poles. **c,** The up-directed field. The helical polarization lines rupture the central Hopfion sphere, pushing it toward the equator. The secondary Hopfion/vortex states, streamlined by the helical lines are formed close to the poles.

.

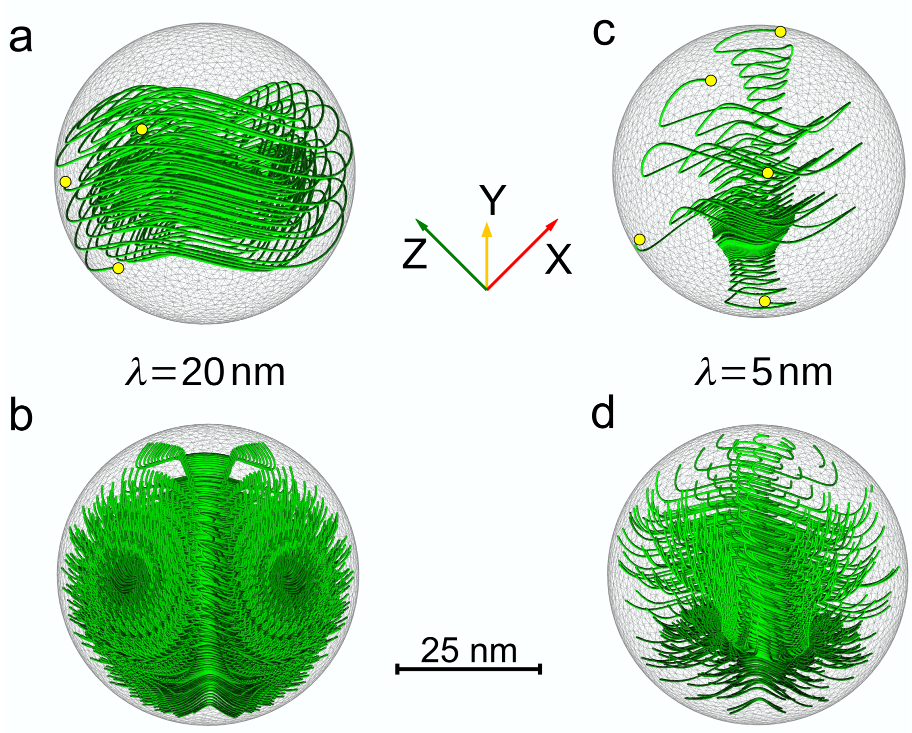

Supplementary Figure 3: Effect of screening on the Hopfion structure in the R = 25 nm PZT particle. a, Slight distortion of the tori texture caused by moderate screening corresponding to the screening length λ = 20 nm. The yellow points depict the spots where polarization lines thrust the surface of the nanoparticle. **b,** A cross section of the nanoparticle demonstrating the details of the tori distortion at λ = 20 nm. The nested tori set transforms into the snail-like convolving configuration. The polarization lines convolve or untwine along the spiral-like paths and creep from torus to torus. **c,** Breakdown of the of the toroidal structure at stronger screening where λ = 5 nm. **d,** A cross section of the nanoparticle at λ = 20 nm demonstrating the tearing of the torus and formation of the vortex-like structure.

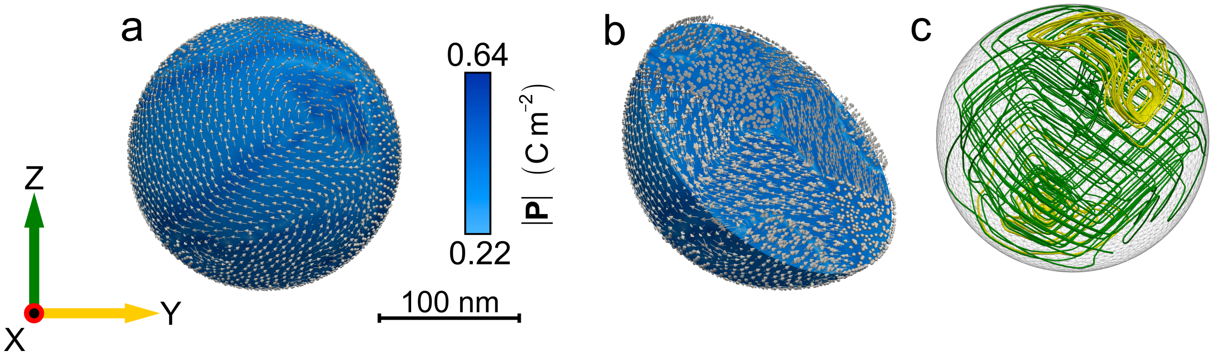

Supplementary Figure 4: The details of the Hopfion formation in the R = 100 nm PZT particle. a, The distribution of the polarization vector, **P**(**r**), over the surface of the nanoparticle. The colour legend shows the amplitude of the polarization. The texture that looks like the seemingly vortex texture now becomes even more shaggy. **b,** The distribution of the polarization vector at the equatorial cut of the nanoparticle. The color legend is the same as in the panel **a,** The texture emerging in the interior appears even more domain-pretentious and, in addition, the new domains with vertical polarization nucleate in the vicinity of the surface. **c,** The analysis of polarization lines reveals that they maintain the Hopfion/vortex structure of Supplementary Figure 1, but both structures become more deformed. In turn, the newly-born domains expose that they are nothing but the little Hopfions, Hopfioninos, that bud off from their parent Hopfion. This is in full accord with the Arnold's theorem that states that the interior of the particle breaks into cells, each hosting the Hopfion fibration (or the vortex texture) of its own.

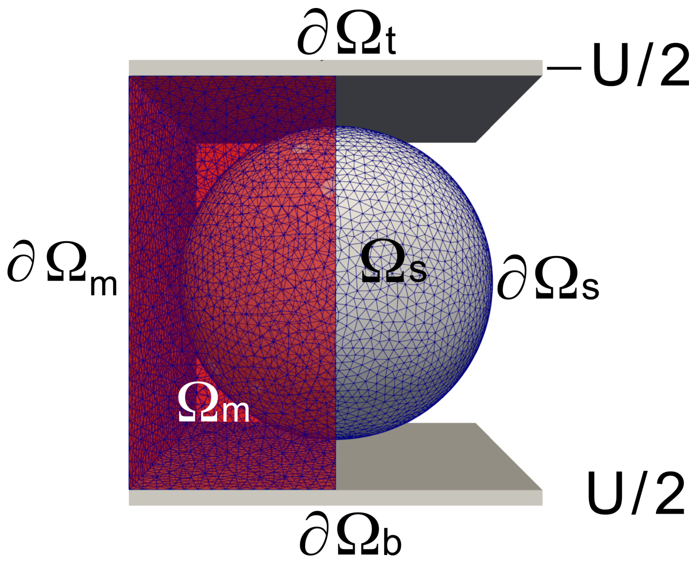

Supplementary Figure 5: Simulation setup.

SUPPLEMENTARY METHODS

Phase-field simulations. The phase-field simulations were performed using the open source FERRET package [1], designed for the open source multi-physics simulation environment MOOSE [2]. We solved time-dependent relaxation equations for the polarization components P_i ($i = 1, 2, 3$)

$$-\gamma \frac{\partial P_i}{\partial t} = \frac{\delta F}{\delta P_i}\,, \tag{1}$$

where F is the total free energy functional, described by equation (2) in the section Methods of the article, which also includes electrostatic and elastic variables, φ and elastic strains u_{ij}, that are found at each relaxation step as solutions of equations (3) and (4) of Methods. The relaxation parameter γ was taken equal to unity since the time scale is irrelevant to the problem.

The geometry of the conceptual setup of the simulated system is shown in Supplementary Figure 5. An entire computational domain, $\Omega = \Omega_s \cup \Omega_m$, consists of the ferroelectric sphere, Ω_s, embedded into the cubic domain of the surrounding media, Ω_m, which in our case is a vacuum. The boundary, $\partial\Omega_m$, of the Ω_m comprises the sides of the cube. The spherical boundary, $\partial\Omega_s$, of Ω_s is the surface of the ferroelectric particle. The parameters of the Ω_m were selected in such a way as to ensure that the size of the cube does not influence the polarization distribution inside the nanoparticle. The finite-element meshes representing the computational domain were generated using the three-dimensional mesh generator software Gmsh [3]. The non-uniform generated meshes were composed of the tetrahedral elements. The first order finite elements of the Lagrange type were used.

To obtain solutions for the relaxation equations we use their variation formulation for the finite-element discretization. The variables at the nodes of the discretization are the polarization components, electric potential, and mechanical displacements. The set of the non-linear algebraic equations resulting from the discretization was solved using the Newton-Raphson method with the help of the generalized minimal residual method (GMRES) for linear systems and additive Schwarz method for the preconditioning [4,5].

The free boundary conditions at the surfaces $\partial\Omega_t$ and $\partial\Omega_b$ were assumed for all variables, except the Dirichlet boundary conditions for the potential, $\varphi(\partial\Omega_t) = -U/2$ and $\varphi(\partial\Omega_b) = U/2$, which were imposed at the top, $\partial\Omega_t$, and bottom, $\partial\Omega_b$, surfaces of $\partial\Omega_m$, to simulate the voltage U, applied to the electrodes.

To ensure the stability of the solution we used the quench from the different initial paraelectric states, assuming distribution of the polarization, with random on-node components P_1, P_2 and P_3 that varied from -10^{-5} to 10^{-5} C m^{-2}. Alternatively, the vortex-like textures, oriented along different crystallographic directions were used as initial conditions. In case of the convergence to different metastable states the texture with the minimal energy was selected as a most stable configuration. In addition, the single- and multi-annealing procedure with respect to the field and temperature in a wide range of these parameters (in particular above the transition temperature), as well as the different finite-element meshes were used. The latter procedure ensures that the Hopfion state is a stable state corresponding to the energy minimum. In order to calculate $P(E)$ characteristics in the ascending/descending fields we used a quasi-stating poling where the polarization distribution at the previous stage was used as an initial condition.

Screening effects. Screening by free carriers allows for the appearance of the depolarization charges at low energy cost. In particular, the presence of surface depolarization charges means that the polarization lines are not tangent to the surface any longer and, therefore, at sufficiently large free carriers density, the Hopfion would finally unwind into uniform polarization. To examine the stability of the Hopfion against the unwinding, one must compare the respective energies of the uniformly-polarized state and the divergenceless Hopfion state. The competition is governed by the contest between the electrostatic energy and the gradient energy of polarization. In the uniform state with zero gradient energy, the depolarization field $E_{\rm d} \simeq P/\varepsilon_0\varepsilon_{\rm b}$ is relevant within the surface layer of the thickness λ having the total volume $4\pi\lambda R^2$. Accordingly, the electrostatic screening energy is $4\pi\lambda R^2\varepsilon_0\varepsilon_{\rm b}E_{\rm d}^2/2$. On the opposite, the electrostatic energy of the Hopfion state is zero because of the absence of the depolarization charges. At the same time, the gradient energy, $(4\pi/3)R^3G(P/R)^2$ (G is a combination of coefficients $G_{\rm ijkl}$), due to the polarization gradient $\sim P/R$ comes from the entire volume. Now the energy condition favoring Hopfion unwinding assumes the form $\lambda < \varepsilon_0\varepsilon_{\rm b}G/R$, which for the nanoparticle with $R = 25\,$nm implies $\lambda < 10^{-4}\,$nm. This simple estimate agrees with ab-initio simulations of polarization distribution in 5-10 nm of nanodots of PZT [6] which show vortex states for nearly all, except short-circuiting, screening conditions and also with numerical calculations of nano-domains in uniaxial ferroelectric $CuInP_2P_6$ [7] where it was ruled that the screening mechanism affects the nano-domain structures only in the case where the enveloping material is made out of the metal in which the screening length is less than 0.05 nm.

SUPPLEMENTARY REFERENCES

[1] Mangeri, J. *et al.* Topological phase transformations and intrinsic size effects in ferroelectric nanoparticles. *Nanoscale* **9**, 1616–1624 (2017).

[2] Gaston, D., Newman, C., Hansen, G. & Lebrun-Grandie, D. Moose: A parallel computational framework for coupled systems of nonlinear equations. *Nucl. Eng. and Design* **239**, 1768–1778 (2009).

[3] Geuzaine, C. & Remacle, J.-F. Gmsh: A 3-d finite element mesh generator with built-in pre-and post-processing facilities. *Int. Journ. for Numerical Methods in Engineering* **79**, 1309–1331 (2009).

[4] Balay, S. *et al.* PETSc users manual. Tech. Rep. ANL-95/11 - Revision 3.8, Argonne National Laboratory (2017).

[5] Balay, S., Gropp, W. D., McInnes, L. C. & Smith, B. F. Efficient management of parallelism in object oriented numerical software libraries. In Arge, E., Bruaset, A. M. & Langtangen, H. P. (eds.) *Modern Software Tools in Scientific Computing*, 163–202 (Birkhäuser Press, 1997).

[6] Ponomareva, I., Naumov, I. & Bellaiche, L. Low-dimensional ferroelectrics under different electrical and mechanical boundary conditions: Atomistic simulations. *Phys. Rev. B* **72**, 214118 (2005).

[7] Eliseev, E. A. *et al.* Labyrinthine domains in ferroelectric nanoparticles: Manifestation of a gradient-induced morphological transition. *Phys. Rev. B* **98**, 054101 (2018).
