# Supplementary material for: Hopfions emerge in ferroelectrics
Source: Nat Commun. 2020 May 15;11:2433. doi: 10.1038/s41467-020-16258-w (PMC7229001; doi:10.1038/s41467-020-16258-w)
Supplement: Supplementary file 3 — Description of Additional Supplementary Files [file 41467_2020_16258_MOESM3_ESM.pdf]

Description of Additional Supplementary Files

Supplementary Movie 1:

Hopfion-assisted switching. The video illustrates the evolution of the polarization configuration upon the system passing the polarization switching displayed on the hysteresis loop for the strongly densified nanoparticles with h=1 nm shown in Fig. 4e of the main text and going through the sequence of topological phase transitions.
